# Relative Validity and Reproducibility of a Dietary Screening Tool in Nigerian Health Care

**DOI:** 10.1016/j.cdnut.2024.104459

**Published:** 2024-09-20

**Authors:** Nimisoere P Batubo, Carolyn I Auma, J Bernadette Moore, Michael A Zulyniak

**Affiliations:** 1 Nutritional Epidemiology Group, School of Food Science and Nutrition, University of Leeds, Leeds, United Kingdom; 2 School of Medicine, Faculty of Medicine and Health, University of Leeds, Leeds, United Kingdom; 3 Food, Nutrition and Health, University of British Columbia, Vancouver, British Columbia, Canada; 4 Institute of Systems, Molecular and Integrative Biology, University of Liverpool, Liverpool, UK

**Keywords:** dietary assessment, food frequency questionnaire, Nigeria, validity, reproducibility, 24-h dietary recalls, noncommunicable diseases, hypertension

## Abstract

**Background:**

Hypertension and cardiovascular disease burden are rising rapidly in Nigeria. This trend is partly attributed to a transition from healthy to unhealthy dietary patterns. However, health care professionals lack a dietary screening tool to assess patient dietary intake and offer personalized dietary advice.

**Objectives:**

We aimed to develop and validate a food frequency questionnaire (FFQ) that can quickly and accurately assess regional dietary intake for use by health care professionals in a hospital setting in Port Harcourt, Nigeria.

**Methods:**

We recruited 58 patients from a single hospital in Nigeria. The FFQ was administered at baseline and again after 3 wk. To evaluate the validity of the FFQ, we used 3 repeated and nonconsecutive 24-h dietary recalls (24DR) as a reference method. Spearman rank correlations, Wilcoxon signed-rank tests, cross-classification, intraclass correlation coefficients (ICCs), and Bland–Altman analysis were performed in R software version 4.3.1 to assess the relative validity and reproducibility.

**Results:**

The mean correlation coefficient (*r*_s_) between the FFQ and 24DR was 0.60 (*P* < 0.05), and ranged from 0.20 to 0.78. The Wilcoxon signed-rank tests indicated no significant differences in the 19 food groups queried (*P* > 0.05), except for fats and oils (*P* < 0.05). The exact agreement for classifying individuals into quartiles ranged from 17% for salt to 88% for processed meats and alcoholic drinks, with 90% of individuals classified into the same or neighboring quartile. Additionally, the Bland–Altman analysis demonstrated acceptable agreement, with >96% of observations within the acceptable limits of agreement for all food groups. For reproducibility, the ICC ranged from 0.31 for stew to 0.98 for fruit, with an mean ICC of 0.77 between the FFQs delivered 2 wk apart.

**Conclusions:**

Our results support the use of the FFQ as a valid and reliable tool for ranking intakes of certain food groups among patients in a hospital setting in Nigeria.

The trial was registered at clinicaltrials.gov as NCT05973760.

## Introduction

Hypertension is a leading modifiable risk factor for cardiovascular disease (CVD), which is responsible for over 10 million deaths worldwide [[1], [2], [3]]. The highest hypertension burdens exist in low-income and middle-income countries, with over 30% of adults affected in some African regions [4,5]. In Nigeria, specifically, hypertension prevalence has more than doubled since 1990, from 11.4% to 36.1% in 2020, with just over a quarter of hypertensive adults achieving blood pressure control [6,7]. Poor diets, that is, unhealthy dietary patterns, are a predominant modifiable risk factor for CVDs and hypertension globally, including in sub-Saharan Africa, accounting for 8 million deaths and responsible for 7 million CVD-related deaths globally in 2021 [2,8].

The rising prevalence of hypertension and other noncommunicable diseases (NCDs) in Nigeria is partly attributed to the rapid dietary transition from healthy to unhealthy dietary patterns, characterized by high intakes of salt, unhealthy fats, refined sugars, ultraprocessed foods, meats, and alcohol, coupled with low fruit and vegetable, whole grain, and nut consumption [9,10]. These unhealthy patterns are often associated with increased risk of hypertension, CVD, certain cancers (eg, colorectal), and other NCDs [8,[11], [12], [13]]. Previous research has highlighted a significant association between high consumption of diets rich in dietary salt, red meat, processed foods, fast foods, fried foods, dietary fat, and alcohol and an elevated risk of hypertension with mean overall risk increase of 1.42 not only in Nigeria but also in various West African countries [10,14,15]. The average daily sodium intake in Nigeria ranges from 9 to 12 g, which exceeds the WHO’s recommended limit of 5 g [16]. This highlights dietary optimization as a crucial component of population level and clinical hypertension prevention strategies globally, including in Nigeria.

Improving diet quality across a population can significantly reduce the prevalence of hypertension and other related NCDs, underscoring the importance of effective dietary assessment and counseling in health care settings [17]. Guidelines and recommendations, including those from the Nigerian National Strategic Plan of Action for Nutrition, the Centre for Disease Control, the United States Preventive Services Task Force, and the American Heart Association, emphasize the need for dietary assessment and counseling by health care professionals in clinical practice [[18], [19], [20], [21]]. However, in Nigeria, health care professionals lack quick and validated dietary screening tools that capture Nigerian foods to assess dietary intake and inform nutritional strategies to manage disease risk.

Dietary assessment tools such as food frequency questionnaires (FFQs), food recalls, and food records are widely accepted field methods for estimating dietary intake in epidemiologic studies, including Nigeria [22,23]. For instance, the Nigeria General Household Survey collects comprehensive dietary data from a representative sample of households, but its 7-d dietary assessment is not validated or tailored for examining the role of diet in NCDs [12,24]. To ensure the relevance and validity of the FFQs to the specific population, it is crucial to develop and validate region-specific, culturally sensitive tools that accurately assess dietary intake in the studied population. For example, Samson et al. [12] developed and validated a semiquantitative FFQ to assess regional diet in a cancer population in Southwest Nigeria among 68 participants. Similarly, Bigman and Adebamowo [25] conducted a validation study for a semiquantitative FFQ among 205 Nigerian adults. Although these FFQs are effective and valid for ranking common foods in Nigerian research settings, they are time consuming and have not been designed for clinical settings.

Therefore, we developed and assessed the relative validity and reproducibility of 28-item FFQ to support health care professionals in a single hospital setting in Nigeria to assess the intake of common food groups among adult patients [26]. By evaluating the reproducibility, we aimed to enhance the FFQ’s applicability in preventing and managing NCDs and provide crucial insights for implementing the FFQ in a Nigerian hospital setting.

## Methods

### Study design and setting

This was a single-center, retrospective study using qualitative approaches to assess dietary intake and evaluate the agreement between the food group intake estimated by the FFQ and repeated 24-h dietary recalls (24DRs). We sought to assess the relative validity and reproducibility of a newly developed tailored dietary screening tool consisting of 28 questions on food item intake that we aim to incorporate into routine clinical practice in Nigerian hospitals to identify adults at high risk of hypertension. The investigation was conducted at the Internal Medicine and Family Medicine Department outpatient clinics of Rivers State University Teaching Hospital (RSUTH) in Port Harcourt, Rivers State, Nigeria. The study protocol underwent review by 2 ethics boards. First, it was submitted to the Business, Earth & Environment, Social Sciences (AREA FREC) Committee at the University of Leeds, Leeds, United Kingdom, on the 21 March, 2023. Subsequently, it was presented to the RSUTH Research Ethics Committee in Port Harcourt, Nigeria, on 20 March, 2023. Final approvals were granted with the following reference numbers: 0484 on 28/04/2023 and RSUTH/REC/2023316 on 30 March, 2023, respectively. The trial was duly registered at clinicaltrials.gov as NCT05973760.

### Development of the FFQ

The development of the FFQ was divided into 5 major sections: First, we conducted a systematic review and meta-analysis to synthesize existing evidence on the association between common diets, foods, and nutrition and risk of hypertension in West Africa, including Nigeria. The findings identified 6 major food groups significantly associated with hypertension in West African countries, which informed the development of a simple FFQ [10]. Second, we created a comprehensive food list comprising 180 common food items based on the evidence from the systematic review and meta-analysis, as well as guidelines from the Nutritional Guidelines on the Prevention of Noncommunicable Diseases of Nigeria and Ghana [27,28]. These items were representative of the regional diets and relevant to hypertension. Third, using the West African Food Composition Table of 2019 [29], we categorized these 180 common food items into a 28-question FFQ with 26 food groups. These food groups encompassed various foods such as fruit, vegetable, fiber-breakfast cereals, rice and pasta, beans, yam and potatoes, fried or fast foods, whole meat, white meat, processed meat, sugary fizzy drinks and fruits, diet nonalcoholic drinks, tea and coffee, soups and stew (fatty soups, vegetable soups, draw soups, native soups, and stews), nuts and seeds, dessert and sweets, fats and oils, salt, and milk and milk-based beverages (Supplemental Table 1), with participants asked to choose their frequency of over the past month. Portion size was not included to simplify the use of the FFQ in hospital settings. Finally, we conducted a feasibility trial and qualitative assessment involving a diverse group of Nigerian adult patients (*n* = 66) from the 4 major ethnicities (ie, Ijaw, Yoruba, Hausa, and Igbo) and health care professionals (*n* = 35) in a hospital setting to trial and review the food lists and gather feedback on their perspectives and experiences using the FFQ. The results of the feasibility trial demonstrated promising evidence from both patients and health care professionals. The feedback from patients and health care professionals was used to refine the FFQ (Supplemental Table 2) [26].

### Sample size

The primary measure for assessing the agreement between the FFQ and 24DRs was the correlation coefficients between these 2 methods. Previous validation studies investigating the correlation between FFQ and 24DR have demonstrated good agreement, with correlation coefficients (*r*_s_) ranging from 0.3 to 0.7 [[30], [31], [32], [33]]. A moderate *r*_s_ of 0.5 is typically considered a strong indicator of correlation [34]. Therefore, the correlation coefficient of 0.5 was used to estimate the required sample size. Using G∗Power software, we estimated the sample size needed to achieve a statistical power of 0.8, with a 95% CI and a 2-tailed α level of 0.05 [35]. The calculation determined that a minimum of 29 participants would be required. To accommodate an anticipated dropout rate of 20% and address any potential missing or incomplete data, we set the target sample size at 50 participants [36,37]. This sample size ensures sufficient power to detect meaningful correlations between the FFQ and 24DR, enhancing the validity and reliability of our study findings.

### Eligible participants

Our study enrolled adult patients visiting the RSUTH for routine medical care between the ages of 18 and 70 y, including men and women who had been residing in Nigeria for ≥2 years at the time of the study and possessed proficiency in reading, writing, and communicating in English. The complete list of inclusion and exclusion criteria is presented in Table 1.

**TABLE 1:** Participant’s inclusion and exclusion criteria.

Inclusion criteria	Exclusion criteria
Age between 18 and 70 y	Individuals <18 y or > 70 y of age
Men and women	Pregnant or breastfeeding women or those intending to become pregnant
Hypertensive or nonhypertensive individual	Diagnosis of other chronic diseases such as cancer, diabetes, renal failure, endocrine diseases, and previous and recent incidence of cardiovascular disease and stroke
Individuals who have been residents in Nigeria for the past 2 y	Individuals who have been resident in Nigeria for shorter than 2 y
Ability to read, write, and communicate over the phone in English	Individuals on dietary restriction or recent changes to their diet or food
Individuals who gave their consent to participate	Individuals who did not give their consent to participate or are currently enrolled in other studies

### Participant recruitment and informed consent

Participant recruitment occurred over 4 wk in July 2023 during regular clinic visits. Eligible participants were recruited through a nonprobability convenience sampling method. This process was facilitated through strategically placed recruitment posters within the hospital premises, referrals from health care professionals, and morning briefing sessions at the outpatient clinics of the Internal Medicine and Family Medicine Departments of RSUTH. Patients expressing interest in the study were screened for eligibility using a structured questionnaire (Table 1). Subsequently, eligible patients were categorized into either the hypertension or nonhypertension groups. Before participation, each participant received and reviewed a simplified version of the participant information sheet. They had the opportunity to address any queries or concerns with the study personnel, ensuring their consent to participate was voluntary and fully informed. All patients provided written informed consent before participating in the study. The study adheres to CONSORT guidelines for reporting clinical trials [38].

### Data collection

#### Dietary intake assessment

##### Reproducibility

**FIGURE 1:**
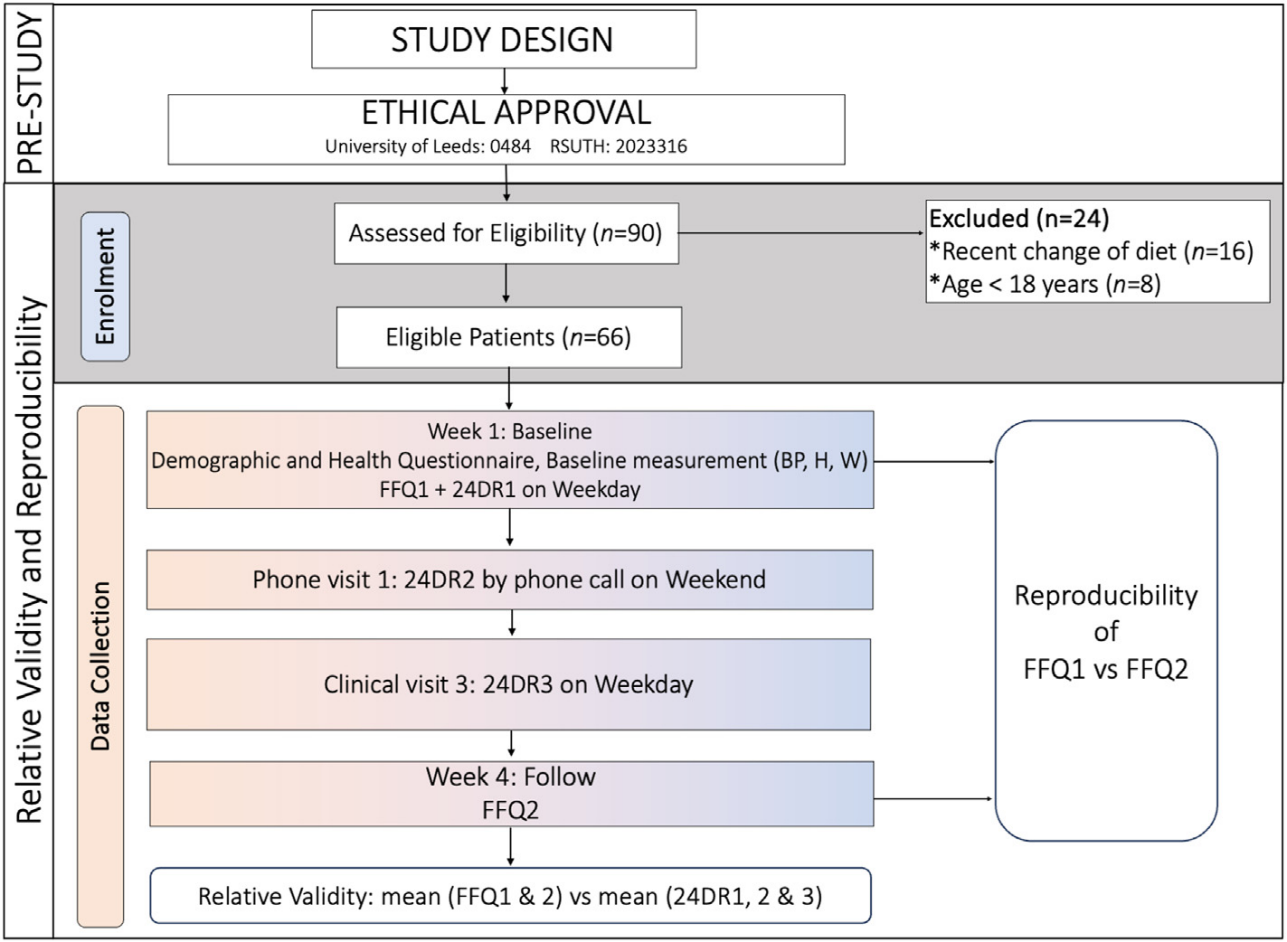
Study design, patient recruitment, enrolment, and data collection flowchart. BP, blood pressure; FFQ1, first food frequency questionnaire; FFQ2, second food frequency questionnaire; H, height; W, weight; 24DR1, first 24-h dietary recall; 24DR2, second 24-h dietary recall; 24DR3, third 24-h dietary recall.

We used the FFQ to assess patients’ food intake for the past month. We administered the FFQ at baseline and followed up 3 wk later between August and September. At the first clinic visit, in week 1, eligible consenting patients completed the first self-administered Food Frequency Questionnaire (FFQ1), and in the fourth clinic week, the study patients completed the FFQ for the second time (FFQ2). This approach aligns with similar studies in the field that have evaluated FFQ’s reproducibility within intervals of 2–4 wk [39,40]. For each food item, participants were asked about the frequency of consumption over the past month, with response options ranging from rarely or never, 1–2 times/wk, and 3–5 times/wk to daily and more than once per day (Supplemental Table 1). To evaluate reproducibility, we compared the food intake assessed by FFQ1 and FFQ2 at the first and fourth visits, 3 wk apart (Figure 1).

##### Relative validity

To evaluate the relative validity of the FFQ, 3 repeated and nonconsecutive 24HDRs were conducted as a reference method within 1 week of obtaining the first FFQ data. The first recall (24DR1) was conducted at baseline on a weekday, the second recall (24DR2) was conducted by phone to collect the patients’ food intake on a weekend day, and the third recall (24DR3) on a weekday (Figure 1) with an interval of 2 days between each recall using the multiple-pass method [[41], [42], [43]]. This approach aimed to account for the day-to-day variation in dietary intake, avoid recall bias, and ensure independence of each day’s dietary intake. Throughout the recalls, detailed descriptions of all foods, snacks, and beverages consumed in the preceding 24 h were recorded, including the amounts of foods consumed, cooking methods, and brand names (where possible). The 24DR were conducted by trained nutritionists. However, as our FFQ was designed to assess the frequency of food group intake without considering portion sizes, we did not use the portion size data from the 24DR in the validity assessment of the FFQ. Instead, we focused on comparing the frequency of food group consumption reported in the FFQ with the occurrences of these food groups identified in the 24DR. This approach ensured that our FFQ remained a simple and practical tool for clinical use, aimed at quickly assessing dietary patterns and supporting personalized dietary advice without the complexity of portion size estimation.

#### Physical and anthropometric measurements

Sociodemographic, clinical, and medical health data were collected from the patients at the first clinic using a structured questionnaire. The eligible consenting patients completed sociodemographic and health status questionnaires and underwent baseline assessments, including height, weight, and blood pressure measurements (Figure 1). The height and body weight were measured twice using a standard stadiometer (model number: DG2301, China), and the BMI (in kg/m^2^) was calculated based on the values from the height and weight using the formula BMI = body weight/height squared. The participant’s blood pressure was recorded twice in the nondominant arm using an automated mercury sphygmomanometer (model number: ZK-BB68; Shenzhen, China)**.**

### Data analysis

#### Data preparation

Dietary data from the FFQ1, FFQ2, and the 3 repeat 24DR1, 24DR2, and 24DR3for each participant were anonymized and entered into a Microsoft Excel spreadsheet with quality control measures. Data entry was done in duplicate and verified by a third reviewer. The frequencies of intakes reported in FFQ1 and FFQ2 were converted into quantitative values (intakes per day) by multiplying the average intake per week and then dividing by 7, following a similar approach used by Fatihah et al. [44]. For example, a frequency of intake of 3–5 times/wk was converted to 0.57 intake/d [(3+5)/2 ÷ 7 d]. The salt intake assessed by the FFQ was coded numerically as 1 for never or rarely, 2 for sometimes, 3 for usually, and 4 for always. Similarly, we calculated the mean frequency of intake (intakes per day) from the three 24-h recalls by comparing the reported frequency of food group intake from the FFQ with the occurrences of these food groups identified in the 24-h recall. This was done to ensure we had a consistent number of food groups for assessing relative validity.

The food intake data from the FFQ and 24DR data were aggregated into 20 major food groups based on their similar physiologic effects and risk of hypertension (Supplemental Table 2). The mean intake from FFQ was calculated by combining the data from both administrations (FFQ1 and FFQ2) following a similar approach used by other FFQ validation studies [39,40,45]. Similarly, the mean intake from recalls (24DR) was computed based on the 3 nonconsecutive recalls. This approach aims to minimize bias and day-to-day variability, according to Rutishauser [46]. The mean for each group between the FFQ and recall were used for the relative validity and reproducibility. The dietary data and their corresponding mean differences between the FFQ and 24DR were tested for normality using the Shapiro–Wilk and Kolmogorov–Smirnov tests with inspection of the histogram [47,48]. Since the data were not normally distributed, nonparametric methods were used for the analysis. The results from this study were reported as mean, median and IQR for continuous data and count (*n*) and percentages (%) for categorical data. *P* values of <0.05 were considered statistically significant. All analyses were performed using an R computing environment (version 4.3.1) [49]. The statistical analyses were performed in 2 phases.

#### Relative validity

In the first phase, we assessed the relative validity of the FFQ by evaluating the agreement between the mean intake from the FFQ1 and FFQ2 and the mean intake from the 24DR following a similar approach used by previous studies [40,45]. Several methods were used. First, Spearman rank correlation was used to compare the frequency of intakes from the FFQ with those from the 24DR. A positive correlation coefficient (*r*_*s*_) above 0.3 indicated a good correlation [50]. Second, the Wilcoxon signed-rank test was used to compare the difference between the mean intake from the FFQs and 24DRs for each food group. A *P* value of >0.05 indicated no statistically significant difference and good agreement between the 2 methods [51,52]. Third, we used cross-classification to classify the intakes into quartiles by 2 methods (FFQ and 24DR) and calculate the proportion of exact agreement (same quartile), adjacent agreement (deviation by 1 quartile), and gross misclassification (disagreement by 3 quartiles). Finally, the Bland–Altman analysis and plots were used to assess the level of agreement and whether differences between FFQ and 24DR estimates were dependent on the magnitude of measurements [53]. We then plotted the mean intake difference (FFQ − 24DR) against the mean of the 2 measures [(FFQ + 24DR)/2] for each food group. An acceptable level of agreement was defined as differences in means falling within the range of ±3 standard deviations (SDs) [54]. The relative differences (%) within this range were also calculated to quantify the proportion of agreement.

#### Reproducibility

In the second phase, we assessed the reproducibility of the FFQ by comparing the frequency of intake from FFQ1 and FFQ2 administrations. First, we evaluated the strength and association of the FFQ1 and FFQ2 using Spearman rank correlations. Second, we assessed the agreement and consistency between the food groups in the 2 FFQ administrations using intraclass correlation coefficients (ICCs). The ICC values were calculated using a single rating, absolute agreement, and 2-way mixed-effects model [55]. ICC values above 0.6 were considered evidence of good reproducibility between the 2 FFQ administrations [50]. Finally, the ranking agreement between the FFQ1 and FFQ2 was evaluated using the Wilcoxon signed-rank test, and a *P* value of >0.05 was considered to indicate a good agreement between the 2 administrations.

## Results

### Participant characteristics

**FIGURE 2:**
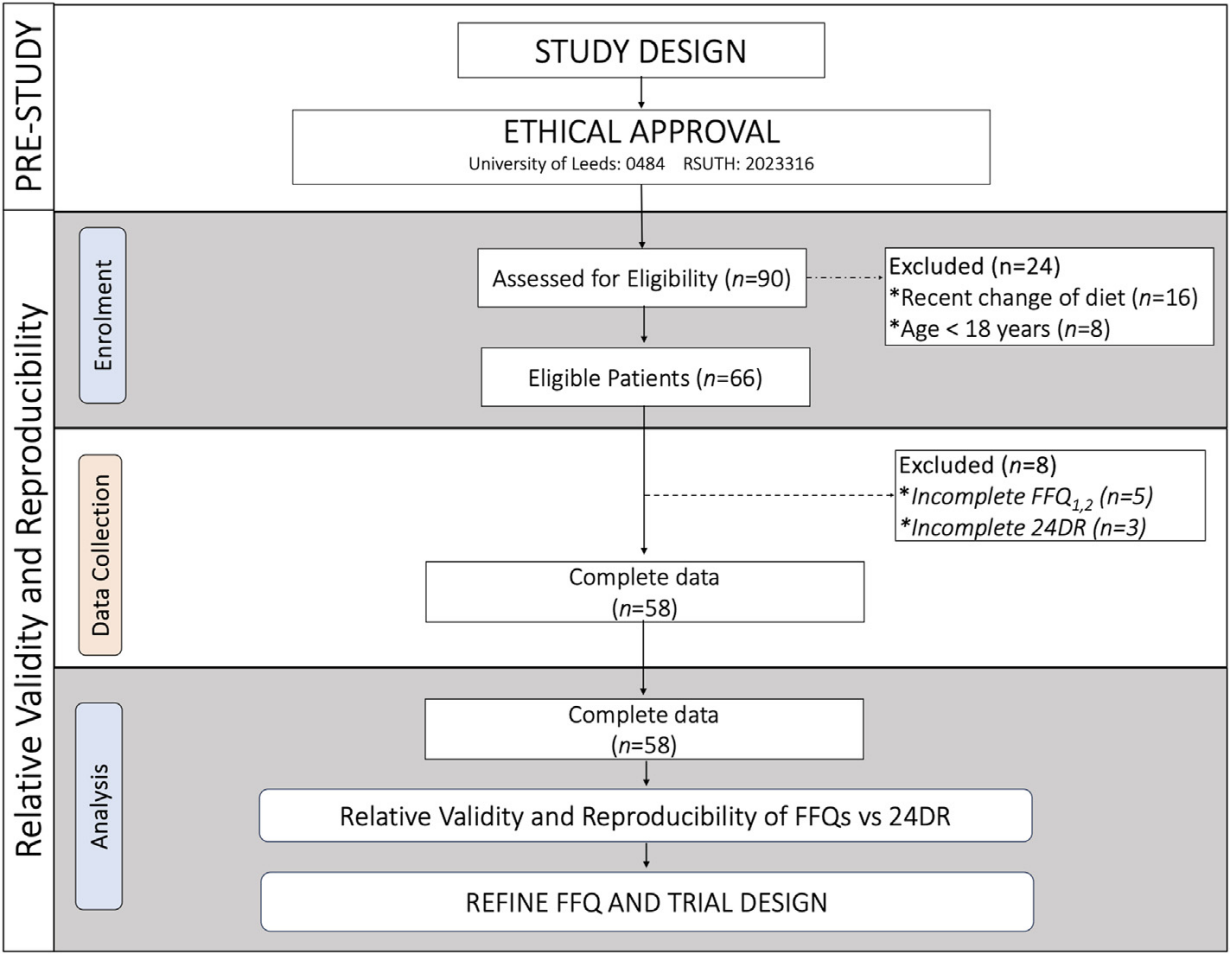
Participant selection and sequence of assessments flowchart. FFQ, food frequency questionnaire; 24DR, 24-h dietary recall.

A total of 90 patients indicated an interest in the study. Of these, 66 met the inclusion criteria and consented to participate in the study. Of the 66 eligible consenting patients, 58 completed the study protocol, and their data were included in the final data analysis (Figure 2). The overall mean age was 42.6 ± 11.9 years, with hypertensive participants being older, on mean 46.4 ± 10.1 years, than nonhypertensive participants with a mean age of 38.7 ± 12.4 years. The majority of participants were females (69%). The distribution of participants by ethnicity indicated that Ijaw participants made up 31%, Hausa participants comprised 17%, Igbo participants accounted for 28%, and Yoruba participants represented 24% (Table 2). Over two-thirds (69%) had a university or postgraduate education. A family history of hypertension was reported by 55.2% (Table 2). A considerable proportion of participants had experienced hypertension for >5 y (41.4%), but only 55.2% reported using antihypertensive medications. Participants with hypertension, on average, appeared to be heavier (83.8 kg compared with 75 kg), with more presenting with obesity (BMI: 32.1 ± 6.4 kg/m^2^) than those who did not have hypertension (26.9 ± 6.8 kg/m^2^). Similarly, participants with hypertension, on average, had higher systolic blood pressures (159.0 ± 16.9 mm Hg compared with 121.0 ± 11.7 mm Hg) despite a high percentage using antihypertensive medications (Table 2).

**TABLE 2:** Sociodemographic, anthropometric, and clinical characteristics of participants.

Characteristics	Overall (*n* = 58)	Nonhypertensive (*n* = 29)	Hypertensive (*n* = 29)
Age (y)	42.6 ± 11.9	38.7 ± 12.4	46.4 ± 10.1
Sex			
Male	18 (31.0)	9 (31.0)	9 (31.0)
Female	40 (69.0)	20 (69.0)	20 (69.0)
Ethnicity			
Ijaw	18 (31)	8 (28)	10 (35)
Hausa	10 (17)	6 (20)	4 (14)
Igbo	16 (28)	7 (24)	9 (30)
Yoruba	14 (24)	8 (28)	6 (21)
Education			
Primary	2 (3.5)	1 (3.5)	1 (3.5)
Secondary	12 (20.7)	3 (10.3)	9 (31.0)
High school	4 (6.9)	2 (6.9)	2 (6.9)
University	26 (44.8)	14 (48.3)	12 (41.4)
Postgraduate	14 (24.1)	9 (31.0)	5 (17.2)
Family history of hypertension			
Yes	32 (55.2)	19 (65.5)	13 (44.8)
Years of hypertension			
<1	9 (31.0)	None	9 (31.0)
1–5	8 (27.6)	None	8 (27.6)
>5	12 (41.4)	None	12 (41.4)
Antihypertensive medications use			
Yes	16 (55.2)	None	16 (55.2)
No	13 (44.8)	None	13 (44.8)
Height (m)	1.7 ± 0.1	1.7 ± 0.1	1.6 ± 0.1
Body weight (kg)	79.4 ± 17.2	75.0 ± 15.4	83.8 ± 18.1
BMI (kg/m^2^)	29.5 ± 7.1	26.9 ± 6.8	32.1 ± 6.4
Blood pressure			
SBP (mm Hg)	140.3 ± 23.9	121.0 ± 11.7	159.0 ± 16.9
DBP (mm Hg)	87.4 ± 17.3	75.4 ± 9.7	99.3 ± 14.8

Data are presented as *n* (%) or mean ± SD.

Abbreviations: DBP, diastolic blood pressure; SBP, systolic blood pressure.

### Dietary intake assessment

The mean and median intakes per day were similar between the 2 dietary assessment methods for all food groups (Table 3). The mean fold differences varied from 0.28 for fats and oils to 1.25 for yam and potatoes, indicating either overestimation or underestimation of the FFQ compared with the 24DR. However, it is important to note that these findings were not statistically significant for 19 food groups (all *P* > 0.05), except for fats and oils (*P* < 0.05). Overall, the mean fold differences indicate that there is generally good agreement between the intake estimated by the FFQ and the 24DR across most of the food groups (*n* = 19).

**TABLE 3:** Mean daily intakes estimated by the Food Frequency Questionnaire and the 24-h dietary recall.

Food group (intakes/d)	FFQ			24DR			Mean fold difference (FFQ/24DR)		
	Mean	Median	IQR	Mean	Median	IQR	Mean	95% CI	*P*
Fruits	0.38	0.21	0.57	0.35	0.33	0.67	1.09	0.82; 1.41	0.288
Vegetables	0.48	0.21	0.36	0.46	0.39	0.36	1.05	0.94; 1.19	0.193
Grains	0.40	0.33	0.19	0.42	0.44	0.20	0.96	0.84; 1.10	0.741
Beans and lentils	0.33	0.21	0.36	0.34	0.33	0.26	0.99	0.82; 1.12	0.723
Meat	0.55	0.50	0.30	0.61	0.50	0.29	0.90	0.80; 1.00	0.975
Processed meat	0.16	0.00	0.21	0.14	0.00	0.00	1.19	0.95; 1.59	0.069
Fish and seafoods	0.62	0.39	0.58	0.54	0.33	0.59	1.16	0.96; 1.36	0.058
Eggs	0.32	0.21	0.36	0.31	0.33	0.67	1.04	0.85; 1.28	0.365
Fried or fast food	0.23	0.21	0.37	0.29	0.33	0.33	0.80	0.62; 1.03	0.954
Yam and potatoes	0.30	0.21	0.18	0.25	0.33	0.33	1.25	0.88; 1.85	0.136
Soups	0.35	0.32	0.22	0.33	0.33	0.23	1.08	0.96; 1.20	0.096
Stew	0.45	0.39	0.36	0.42	0.33	0.34	1.09	0.92; 1.27	0.164
Nuts and seeds	0.46	0.29	0.36	0.44	0.33	0.67	1.06	0.86; 1.29	0.298
Desserts and sweets	0.19	0.00	0.21	0.21	0.00	0.33	0.96	0.60; 1.48	0.615
Soft drinks	0.22	0.13	0.24	0.19	0.17	0.33	1.15	0.85; 1.57	0.222
Alcoholic drinks	0.03	0.00	0.00	0.04	0.00	0.00	1.00	0.43; 1.57	0.787
Tea and coffee	0.39	0.21	0.47	0.34	0.33	0.67	1.15	0.89; 1.47	0.153
Milk and milk drinks	0.46	0.39	0.36	0.48	0.33	0.34	0.96	0.82; 1.11	0.739
Fats and oils	0.58	0.57	0.74	2.30	2.33	0.92	0.28	0.22; 0.33	<0.001
Salt and seasonings	3.37	4.00	1.00	3.45	3.33	1.33	1.05	0.94; 1.16	0.413

Abbreviations: FFQ, Food Frequency Questionnaire; 24DR, 24-h dietary recall.

### Assessment of relative validity

To assess the validity of the FFQ, we evaluated the relationship between the food group intakes estimated by the FFQ relative to the 24DR. The Spearman correlation coefficients (SCCs; *r*_s_) ranged from 0.20 for fats and oils to 0.78 for vegetables, with an mean correlation coefficient of 0.60 (Table 4). Although weaker positive correlation coefficients (*r*_s_ < 0.3) were found for fat and oils, and salt, most of the food groups (*n* = 15) had a correlation coefficient of ≥0.50, indicating a strong positive correlation between the mean FFQ and mean 24DR (*P* < 0.05). In addition, among the 20 food groups evaluated in the FFQ, 19 food groups had no significant differences (*P* > 0.05) in the mean and median intakes compared with those in the 24DR when a Wilcoxon signed-rank test was applied—the exception was fats and oils (*P* < 0.05) (Table 4). The findings suggest that the FFQ provides comparable rankings and intake estimates for most foods (*n* = 19) with those of 24DR and shows good agreement between the dietary assessment approaches.

**TABLE 4:** Comparison of mean daily intakes between the food frequency questionnaire and the 24-h dietary recalls.

Food group (intakes/d)	Agreement between FFQ and 24DR			Disagreement between FFQ and 24DR		
	*r*_s_	*P*^1^	*P*^2^	Exact (%)	Adjacent (%)	GM^3^ (%)
Fruit	0.65	<0.001	0.748	53	33	14
Vegetables	0.78	<0.001	0.706	50	45	5
Grains	0.64	<0.001	0.042	40	53	7
Beans and lentils	0.64	<0.001	0.632	53	40	7
Meat	0.65	<0.001	0.063	50	43	7
Processed meat	0.74	<0.001	0.215	88	10	2
Fish and seafoods	0.72	<0.001	0.869	62	35	3
Eggs	0.77	<0.001	0.224	28	55	17
Fried or fast food	0.48	<0.001	0.081	52	33	15
Yam and potatoes	0.37	0.004	0.619	45	45	10
Soups	0.66	<0.001	0.361	48	45	7
Stew	0.62	<0.001	0.174	47	47	7
Nuts and seeds	0.71	<0.001	0.862	66	31	3
Desserts and sweets	0.47	<0.001	0.237	64	26	10
Soft drinks	0.65	<0.001	0.806	69	29	2
Alcoholic drinks	0.63	<0.001	0.287	88	12	0
Tea and coffee	0.55	<0.001	0.501	48	38	14
Milk and milk drinks	0.75	<0.001	0.338	67	26	7
Fats and oils	0.20	0.135	<0.001	22	59	19
Salt and seasonings	0.22	0.154	0.968	17	24	59

Abbreviations: FFQ, food frequency questionnaire; *r*_s_, Spearman correlation coefficient; 24DR, 24-h dietary recalls.

1 *P* value of Spearman rank correlation coefficients.

2 *P* value for Wilcoxon signed-rank test of difference.

3 Gross misclassification, disagreement by 3 quartiles.

Additionally, the percentage of participants grossly misclassified by 3 quartiles ranged from 0% for alcoholic drinks to 59% for salt, with an mean of 11% (Table 4). For most food groups (*n* = 15), over 50% of the participants were classified into the same or neighboring quartile. Specifically, the classification of participants into the exact or adjacent quartiles ranges from 10% for dessert and sweets to 88% for processed meat and alcoholic drinks, with an mean exact agreement of 53% and an adjacent agreement of 37% (Table 4). Importantly, 90% of participants were classified in the same or neighboring quartile, indicating a good agreement between the FFQ and 24DR.

**FIGURE 3:**
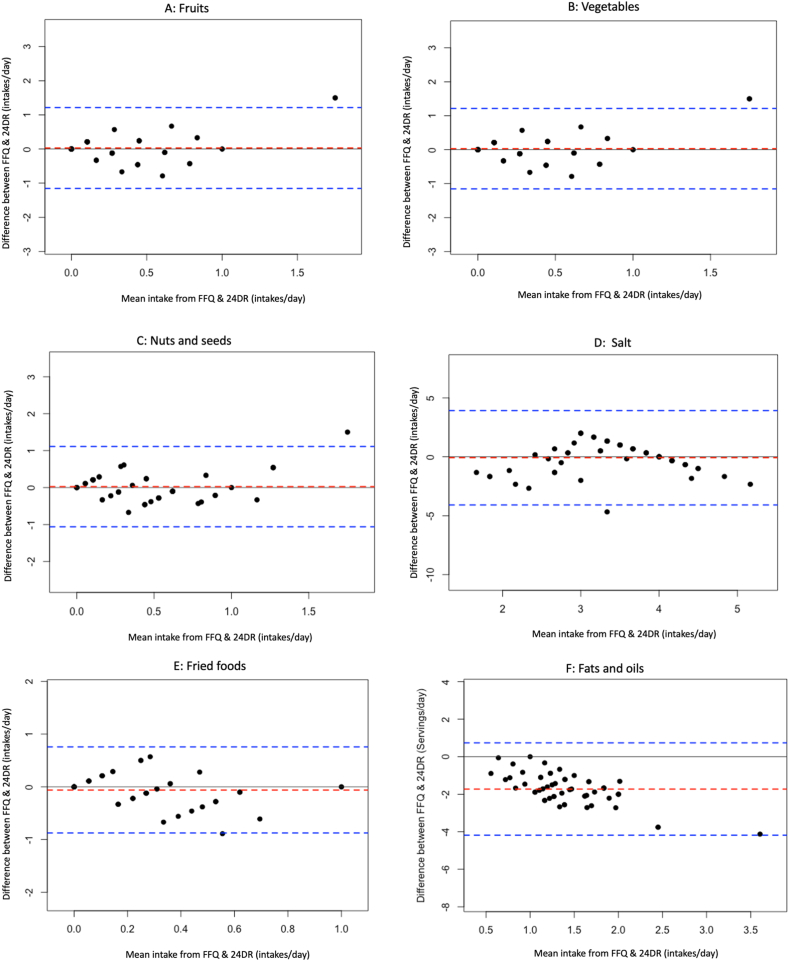
Bland–Altman plots related to food groups identified in the DASH diet: (A) fruit, (B) vegetables, (C) nuts and seeds; (D) salts, (E) fried and fast foods, and (F) fats and oils. Differences in the intake per day of food groups derived from the mean of the 3 repeat 24-h recalls (24DRs) and the mean of the food frequency questionnaire (FFQ) were plotted against the corresponding mean intake per day derived from the 2 methods. Dashed red lines represent the mean difference (bias), and dashed blue lines show the upper and lower 95% limits of agreement (*n* = 58).

Furthermore, the Bland–Altman analysis was used to assess the level of agreement between the FFQ and 24DR (Supplemental Table 3). Figure 3A–F presents the Bland–Altman plots for the 3 healthy food groups of the DASH diet (eg, fruits, vegetables, and nuts and seeds) [56] and 3 less healthy food groups/items identified by our recent meta-analysis of foods associated with hypertension in West African countries, including Nigeria [10] (eg, salt, fried/fast foods, and fats and oils). The plots for the remaining food groups are provided in Supplemental Figure 1. Although moderate bias and wide limits of agreement (LOAs; −4.18 to 3.93) were observed for fats/oils and salt food groups (Figure 3D–F), very limited bias was observed for the majority (*n* = 18) of the food groups where mean differences (bias) ranged from −0.06 intakes/d (meat and fried and fast foods) to 0.08 intakes/d (fish) (Figure 3A–C and Supplemental Figure 1). In addition, the 95% LOA spanned 0.19 to 1.40 intakes/d (upper LOA) and −1.23 to −0.20 intakes/d (lower LOA) for most food groups (*n* = 18), showing reasonable agreement (Supplemental Table 3). A high proportion (>96%) of observations fell within the acceptable LOAs (±3 SD LOA) without increased differences across higher food intake ranges (Supplemental Table 3). In summary, the Bland–Altman analysis and plots suggest a high level of agreement between the FFQ and 24DR for the majority of the assessed food groups (*n* = 18).

### Assessment of reproducibility

Assessing reproducibility between the 2 administrations of the FFQ, Spearman ranked correlation coefficient ranged from 0.38 for yam and potatoes to 0.97 for salt, with an mean correlation coefficient of 0.75, with most food groups (17/20) showing correlation coefficients above 0.60. All correlation coefficients were statistically significant (*P* < 0.001), reaffirming the high level of agreement between the 2 FFQs (Table 5). Additionally, among the 20 food groups evaluated for reproducibility, no significant differences in the mean and median intakes between the FFQ1 and FFQ2 were observed in the Wilcoxon signed-rank test for all food groups (*P* > 0.05) (Table 5). Furthermore, the ICC was used to evaluate the consistency and agreement between the FFQ1 and FFQ2 (Table 5). ICCs ranged from 0.31 for stew to 0.98 for fruit, with an mean ICC of 0.77. The majority of food groups (*n* = 17) had ICC of ≥0.70, which, according to the criteria of Koo and Li [55] and Cade et al. [50], indicates good to excellent reproducibility (Table 5). These findings suggest good reproducibility and consistency in individual rankings and negligible between time points for the FFQ, confirming the test–retest reliability of the FFQ across the food groups evaluated.

**TABLE 5:** Reproducibility on the number of food group intakes per day estimated by repeated administration of the food frequency questionnaire.

Food groups (intakes/d)	FFQ1			FFQ2			Reproducibility (FFQ1 and FFQ2)			
	Mean	Median	IQR	Mean	Median	IQR	*r*_s_	*P*^1^	ICC	95% CI
Fruit	0.38	0.21	0.57	0.39	0.21	0.57	0.90	0.750	0.98	0.96, 0.98
Vegetables	0.49	0.21	0.36	0.46	0.21	0.36	0.84	0.154	0.92	0.88, 0.95
Grains	0.43	0.21	0.57	0.42	0.21	0.57	0.70	0.577	0.72	0.64, 0.79
Beans and lentils	0.32	0.21	0.36	0.33	0.21	0.36	0.91	0.479	0.87	0.80, 0.92
Meat	0.59	0.57	0.79	0.56	0.57	0.79	0.83	0.123	0.85	0.80, 0.90
Processed meat	0.16	0.00	0.21	0.15	0.00	0.21	0.79	0.751	0.87	0.79, 0.92
Fish and seafoods	0.65	0.39	0.79	0.58	0.39	0.79	0.79	0.166	0.80	0.68, 0.88
Eggs	0.31	0.21	0.36	0.33	0.21	0.36	0.85	0.590	0.79	0.66, 0.87
Fried or fast foods	0.26	0.21	0.48	0.22	0.21	0.21	0.76	0.203	0.73	0.59, 0.83
Yam and potatoes	0.29	0.21	0.27	0.32	0.21	0.00	0.38	0.886	0.55	0.34, 0.71
Soups	0.35	0.21	0.36	0.21	0.21	0.36	0.65	0.783	0.75	0.69, 0.80
Stew	0.45	0.39	0.36	0.46	0.21	0.36	0.53	0.867	0.31	0.06, 0.53
Nuts and seeds	0.54	0.21	0.36	0.39	0.21	0.36	0.68	0.099	0.50	0.28, 0.67
Desserts & sweets	0.19	0.00	0.21	0.19	0.00	0.21	0.55	0.746	0.45	0.21, 0.63
Soft drinks	0.23	0.00	0.21	0.21	0.00	0.21	0.87	0.290	0.92	0.89, 0.95
Alcoholic drinks	0.04	0.00	0.00	0.03	0.00	0.00	0.71	0.773	0.94	0.91, 0.97
Tea and coffee	0.38	0.21	0.36	0.39	0.21	0.57	0.73	0.944	0.73	0.58, 0.83
Milk & milk drinks	0.44	0.21	0.36	0.46	0.39	0.36	0.92	1.000	0.95	0.92, 097
Fats and oils	0.57	0.57	0.79	0.57	0.57	0.79	0.62	0.985	0.72	0.57, 0.82
Salt intake	0.83	1.00	0.29	0.81	1.00	0.29	0.97	0.371	0.96	0.94, 0.98

Abbreviations: FFQ1, first food frequency questionnaire administration; FFQ2, second food frequency questionnaire administration; ICC, intraclass correlation coefficient; *r*_s_, Spearman rank correlation coefficient, IQR, interquartile range.

^1^*P* value for the test of the difference between FFQ1 and FFQ2 by Wilcoxon signed-rank test.

1 *P* value for the test of the difference between FFQ1 and FFQ2 by Wilcoxon signed-rank test.

## Discussion

To our knowledge, this study is the first to validate a rapid 28-item FFQ for dietary screening of men and women for high-risk dietary behavior associated with NCDs, including hypertension, in Nigerian hospitals. Our aim was for the tool to be used by health care professionals and patients across Nigeria and West Africa to the following: *1*) estimate dietary intake in routine clinical care; *2*) facilitate discussions of dietary behaviors and cardiovascular health in the hospital; *3*) inform personalized dietary advice for patients at risk or with hypertension; and *4*) empower its citizens to take an active role in preventing and managing NCDs, including hypertension. With the participation of 58 men and women, the FFQ demonstrated good validity and reproducibility for estimating dietary intakes in a Nigerian hospital, compared with 24DR. This validated and rapid FFQ is now called the Nigerian Dietary Screening Tool (NiDST).

### Relative validation

The relative validity of the FFQ was assessed by comparing its agreement with the 24DR. The FFQ demonstrated moderate to strong positive SCCs ranging from 0.20 to 0.78, with the majority of the food groups (*n* = 18), demonstrating moderate to strong positive correlations (*r*_s_ > 0.30), with an overall mean SCC of 0.6. These findings exceed or were consistent with similar findings reported in Nigeria and outside Nigeria. For instance, Bigman and Adebamowo [25], in their validation studies among 205 adult Nigerians, developed a FFQ and food picture book for Nigerian adults to assess its reproducibility and validity compared with 24DRs during different seasons in the year. They reported an overall mean correlation of 0.27. Another validation study conducted by Eghtesad et al. [45] among 978 participants recruited from 7 PERSIAN cohort centers to assess the validity and reproducibility of FFQ, through 26 food group intakes reported SCCs ranging from 0.30 to 0.79 between the FFQ1 and FFQ2, and 24DR [45]. Overall, these findings indicate good agreement and suggest that the FFQ can accurately estimate dietary intake compared with the 24DR.

Moreover, some underestimation and overestimation is expected in all validation studies but must be within an acceptable range. A study by Streppel et al. [57] evaluated the validity of an FFQ against the 24DR among 128 Dutch adults and reported overestimation in 13 of 21 foods by the FFQ. In addition, Steinemann et al. [32] also reported overestimation in 13 of 25 foods compared with a 4-d weighed food record among 56 participants in Germany, with correlation coefficients ranging from 0.09 (soup) to 0.92 (alcohol) with 16 of the 25 food groups having correlation coefficients of <0.50. Our FFQ demonstrated different measures of overestimation or underestimation ranging from 4% to 25% among the food groups assessed compared with previous studies [32,57]. However, these were not statistically significant, indicating a generally good agreement between intake estimated by the FFQ compared with that by the 24DR across most food groups (*n* = 19).

Furthermore, our study reported that 90% of participants were classified into the same or neighboring quartiles when comparing FFQ and 24DR. The mean exact agreement for all food groups was 53%, with an mean gross misclassification of 11% across food groups, indicating how well the FFQ agree with the 24DR in ranking individuals’ dietary intake. These findings are in agreement with other successful validation studies in Australia (*n* = 96 adults) that reported 27%–70% exact agreement and <15% gross misclassification for most food groups [45,58,59]. For example, Eghtesad et al. [45] reported a similar classification of participants, with 51.7% on average correctly classified into the same tertiles for all food groups in the mean intake from FFQ1 and FFQ compared with 24DR with ∼1 in 4 individuals being misclassified in these groups [45]. Additionally, the Bland–Altman method was used to illustrate the level of agreement between the FFQ and 24DR [53]. Although fats and oils and salt were underestimated by the FFQ, as noted in other studies, the majority of the food groups (*n* = 18) assessed in our study demonstrated minimal bias [60]. Indeed, >96% of participants were within acceptable LOAs for the majority of food groups (*n* = 19). These findings align with or exceed the results of previous work, where FFQ validations study among *1*) 130 men with prostate cancer reported similar small mean differences and acceptable agreements across 11 food groups; *2*) 114 Lebanese adults with >80% agreement for the majority of the food groups; and *3*) 205 Nigerian adults with >90% of participants within the LOAs [25,54,61]. Collectively, the results of our study suggest that the validity of our FFQ meets or exceeds the levels of agreement reported by other validation studies and indicates that our culturally appropriate FFQ is well-designed for capturing the dietary intake of men and women in Nigerian populations.

### Reproducibility

The reproducibility of a FFQ is an important attribute for minimizing recall bias in estimating dietary intake with FFQ [46,62]. Our FFQ exhibited commendable reproducibility between the 2 collection points (FFQ1 compared with FFQ2), yielding a strong positive SCC ranging from 0.38 for yam and potatoes to 0.97 for salt intakes with an mean of 0.75 and with SCC that surpassed *r*_s_ > 0.5 for the majority of food groups and ICC, ranging from 0.31 for stew to 0.98 for fruit with an mean of 0.77 and with ICC of >0.70 for the majority of food groups. These findings align with or exceed the results of other FFQ reproducibility studies that reported correlation coefficients between 0.32 and 0.90 and ICC values ranging from 0.65 to 0.98 and agree with current recommended standards for reproducibility between 0.5 and 0.70 [45,50,54,55,61,63,64]. For instance, a similar study conducted in Nigeria by Bigman and Adebamowo [25] among 205 participants reported a mean SCC of 0.39 and a mean ICC of 0.39. According to these criteria, our FFQ demonstrated good levels of agreement between baseline and follow-up dietary intake estimation. These findings suggest the FFQ is well suited for accurately and effectively collecting dietary information and capturing dietary inconsistencies in Nigeria hospitals for clinicians, researchers, and public health professionals.

### Practical application and clinical relevance

The validity and reproducibility of our study dietary data provide compelling evidence to further investigate the implementation and use of our FFQ as a valid NiDST for hospital use to screen and evaluate patient-mediated dietary risk for NCDs, including hypertension. In this study, the NiDST was able to accurately and effectively rank intakes of food groups, including fruits, vegetables, grains, dairy, salt and fats and oil-based foods (soups and stew), to a similar degree of accuracy as 24DR but was able to be completed in <8 min [10,11,26]. The results indicate that the NiDST *1*) is a rapid dietary assessment tool; *2*) can be used in hospital settings; *3*) can effectively identify individuals with high-risk dietary patterns associated with hypertension, diabetes, and certain cancers; and *4*) supports health care professionals to provision personalized dietary advice, education, and support around dietary modification. Therefore, integrating this rapid and validated regionally specific dietary screening tool (NiDST) into primary and tertiary care workflows will be a key step in enabling a systematic approach to dietary intake estimation, monitoring, and counseling in clinical practice to prevent and manage NCDs, including hypertension in Nigeria and other West African countries [26].

### Strengths and limitations

The study highlights the strength and potential of the FFQ—the NiDST—in a hospital setting, along with several limitations. A significant limitation is the qualitative nature of our FFQ, which did not specify food portions, thus preventing the calculation of energy, macronutrient intake, and micronutrient intake. Consequently, our results are limited to frequencies of food group consumption rather than quantitative dietary intake. This design was chosen to develop a quick and simple FFQ suitable for hospital settings, avoiding time constraints and the advanced nutritional knowledge associated required for detailed nutritional data analysis and interpretation. Inherent challenges with both the FFQ and recall, such as potential recall biases and within-person variability in daily intake, could attenuate validation study results [65]. To minimize these limitations, we used a designated professional to perform all 24-h recall evaluations and used 3 repeated multiple nonconsecutive days of recall, including both weekdays and weekends, to capture intraindividual variation. Another limitation is the use of a nonrandom, convenience sample and single-center data collection, which may restrict the generalizability to the broader Nigerian population. However, this approach aligns with the tool’s intended hospital, where feasibility and practicality are prioritized over population representativeness. Additionally, literacy barriers among participants necessitated interviews instead of self-administration, which may potentially influence responses owing to respondent bias or social desirability. Furthermore, testing the NiDST in a relatively small geographic area with diverse cultures may limit its broader applicability of results across more widely diverse hospital settings in Nigeria, a limitation we aim to address in future studies.

Despite these limitations, this study boasts several strengths as follows: *1*) the use of multiple repeated 24DR as the reference method provided detailed participant-informed dietary intake data and enabled assessment of day-to-day variability, thereby strengthening the quality of the reference data; *2*) the use of multiple statistical methods to assess the validity and reproducibility facilitated a comprehensive assessment of the agreement between the FFQ and the 24DR; and *3*) evaluation of the reproducibility or test–retest reliability of the FFQ 3 weeks apart provided insights into the reliability and consistency of the FFQ over time, offering a better measure of habitual dietary habits. Moreover, testing the FFQ within this demographic, hospital, and clinic setting and cultural context (involving the 3 major ethnic groups in Nigeria) for its intended use enhances the tool’s relevance and applicability [26]. Finally, the food list incorporated into the FFQ was informed by evidence from a systematic review and meta-analysis of dietary factors and hypertension risk in West Africa, as well as guidance from the national nutrition guidelines of both Nigerian and Ghana National Nutritional Guideline on Noncommunicable Disease Prevention, Control and Management, and input from stakeholders (eg. patients and health care professionals), ensuring cultural appropriateness to common foods consumed by Nigerian and adapted for use in hospital settings in West African countries [10,[26], [27], [28]].

## Conclusions

This study provides important evidence that the NiDST) has good relative validity and reproducibility for ranking dietary intake of major foods and food groups in a clinical setting, compared with the mean of 3 repeat nonconsecutive 24DRs. Therefore, we offer a valid and reliable NiDST that could help assess common food group intakes among Nigerians, which could empower clinicians, patients, and researchers to take an active role in preventing NCDs, including hypertension, in Nigeria and other West African countries. Further refinements and validation studies of the tool in other regions of Nigeria and on the implementation strategies of the NiDST will improve validity for some food groups.

## Author contributions

The authors’ responsibilities were as follows—NPB, MAZ: designed research; NPB: conducted research and analyzed data; NPB: wrote the paper; MAZ, JBM: provided analytical expertise; MAZ, JBM, CIA: provided critical feedback; NPB: revised the manuscript; and all authors: have read and approved the final manuscript.

## Conflicts of Interest

MAZ reports financial support was provided by Wellcome Trust. NPB reports financial support was provided by Tertiary Education Trust Fund. All other authors report no conflicts of interest.

## Funding

This study was funded by the Tertiary Education Trust Fund (TETFund) of Nigeria. The funders did not have any role in any aspect of this study, including study design, data collection and analysis, decision to publish, or manuscript preparation.

## Data availability

Data described in this manuscript and analytic code will be made available upon request from the corresponding author, pending application and approval.
