# Supplementary material for: Relative Validity and Reproducibility of a Dietary Screening Tool in Nigerian Health Care
Source: Curr Dev Nutr. 2024 Sep 20;8(10):104459. doi: 10.1016/j.cdnut.2024.104459 (PMC11490926; doi:10.1016/j.cdnut.2024.104459)
Supplement: Multimedia component 1 [file mmc1.docx]

**Validity and Reproducibility? of a Culturally Tailored Dietary Screening Tool for Hypertension Risk in Nigerian Healthcare**

**Nimisoere P. Batubo^1^, Carolyn I. Auma^1^, J. Bernadette Moore^1^, and Michael A. Zulyniak^1*^**

^1^ Nutritional Epidemiology Group, School of Food Science and Nutrition, University of Leeds, Leeds, LS2 9JT.

**^*^ Corresponding author:**

[m.a.zulyniak@leeds.ac.uk](mailto:m.a.zulyniak@leeds.ac.uk) (MAZ)

**Trial registration*:*** *ClinicalTrials.gov*: NCT05973760. Registered August 3^rd^, 2023

**Table S1.** Food Frequency Questionnaire

The following questions ask about some foods & drinks you might have during a ‘typical’ week over the past month or so. Do not be concerned if some things you eat or drink are not mentioned. Please tick how often you eat at least ONE portion of the following foods & drinks. Please only put one tick, but answer EVERY line.

**Time started:  :**

| Section 2: Dietary intake information | | | | | | | | | | | | |
| --- | --- | --- | --- | --- | --- | --- | --- | --- | --- | --- | --- | --- |
| S/N | Food group/item | | | Frequency | | | | | | | | |
|  |  |  |  | More than once/ day | | Daily | 3-5 times a week | | 1-2 times a week | | Never/  Rarely | |
| 1 | Fruit  *(such as Cherry, Mango, Oranges, Pineapples, Pawpaw, Bananas, Guavas, Avocado (peas), Grapefruit, Tangerines, Soursop, Butter pear, and others)?* | | |  | |  |  | |  | |  | |
| 2 | Vegetables and Corn (maize)  *(such as Tomatoes, Water leaves, Cabbage, Carrots, Cucumber, Watermelon, Garden eggs, Eggplant, Broccoli, Corn, and others)?* | | |  | |  |  | |  | |  | |
| 3 | Rice and Pasta  *(such as White rice, Spaghetti, Macaroni, and Noodles)?* | | |  | |  |  | |  | |  | |
| 4 | Wheat products  *(such as Semolina and wheat bread, white bread, brown bread)?* | | |  | |  |  | |  | |  | |
| 5 | Fibre-rich breakfast cereals  *(such as Cornflakes, Golden morn, Weetabix, Fruit ‘n Fibre, and others)?* | | |  | |  |  | |  | |  | |
| 6 | Beans  *(such as Boiled beans, porridge beans)?* | | |  | |  |  | |  | |  | |
| 7 | Whole Meat  *(such as Cow meat, Beef, Lamb, Pork, Bush meat and others)?* | | |  | |  |  | |  | |  | |
| 8 | White Meat  *(such as Chicken, Turkey, and others)?* | | |  | |  |  | |  | |  | |
| 9 | Processed Meats  *(such as Suya, Kilishi, Sausages, bacon, corned beef, meat pies, burgers, Gala, and others)?* | | |  | |  |  | |  | |  | |
| 10 | Fish (unbattered) or Whole fish? | | |  | |  |  | |  | |  | |
| 11 | Eggs  *(such as Boiled eggs, fried eggs, and scrambled eggs)?* | | |  | |  |  | |  | |  | |
| 12 | Fried or fast (takeaway) foods  *(such as Fried rice, Jollof rice, Akara, Puff-puff, Pancakes, Chin-chin, Buns, Chips (plantain, yam, and potatoes), Shawarma, Pizza, Moin-moin, fried fish, fried chicken)?* | | |  | |  |  | |  | |  | |
| 13 | Potatoes and Yam  *(such as Boiled yam, Amala, pounded yam, porridge yam, boiled potatoes)?* | | |  | |  |  | |  | |  | |
| 14 | Fatty soups  *(such as Egusi (melon) soup, Banga soup or groundnut soup and others)* and Swallow *(Fufu, Garri, and Eba)?* | | |  | |  |  | |  | |  | |
| 15 | Vegetable soups  *(such as Afang soup, Edikaikong soup, Bitter leaf soup, Efo Riro and others)* and Swallow *(Fufu, Garri, and Eba)?* | | |  | |  |  | |  | |  | |
| 16 | Draw soups  *(such as Okra soup, Ogbono soup, Ewedu soup and others)* and Swallow *(Fufu, Garri, and Eba)?* | | |  | |  |  | |  | |  | |
| 17 | Native soup  *(such as Fisherman soup, white soup, Nsala soup and others)* and Swallow *(Fufu, Garri, and Eba)?* | | |  | |  |  | |  | |  | |
| 18 | Stew (sauce)  *(such as Palm oil stew, vegetable oil stew and others)?* | | |  | |  |  | |  | |  | |
| 19 | Nuts/seeds  (*such as Groundnut, peanut, coconut, and others)?* | | |  | |  |  | |  | |  | |
| 20 | Desserts and sweets  *Ice cream, yoghurt, cheese, sweet biscuits, cakes, chocolate, sweets, and others?* | | |  | |  |  | |  | |  | |
| 21 | Fat and Oils  *(such as Palm oil, Vegetable oil, Groundnut oil, Coconut oil, butter, lard, mayonnaise/salad cream, margarine)?* | | |  | |  |  | |  | |  | |
| 22 | Fizzy (Soft) drinks and fruit juices  *(such as Coke, Fanta, sprite, Pepsi, Betas Malt, Guinness Malt, Maltina, Zobo, Tiger nut, Kunu, 5live, Pineapple juice and others)?* | | |  | |  |  | |  | |  | |
| 23 | Diet Non-alcoholic fizzy drinks  *(such as Diet Coke, diet Fanta, diet sprite, energy drink and others)?* | | |  | |  |  | |  | |  | |
| 24 | Alcoholic drinks  *(such as Beer, wine, gin, whisky, spirit, and others)?* | | |  | |  |  | |  | |  | |
| 25 | Tea and Coffee  *(such as Green tea, Lipton, Nescafe and others)?* | | |  | |  |  | |  | |  | |
| 26 | Milk or milk-based beverages  *(such as Peak, Three Crowns, Dano, Cowbell, Loya Milk, Coast, Hollandia, Nunu, Milo, Ovaltine, Chocomilo, Nesquik, Nutri-C, Viju Milk Drink, and others)?* | | |  | |  |  | |  | |  | |
| 27. | Dietary salt | Frequency | | | | | | | | | |  |
|  |  | Never/ Rarely | Sometimes | | Usually | | | Always | | Don’t know | |  |
| Do you add salt to food while cooking including seasoning cubes (such as Salt, Seasoning (e.g., Maggi, Knorr), Sauce (e.g., Soy sauce, Suya sauce, Fish sauce, Oyster sauce), and others) | |  |  | |  | | |  | |  | |  |
| How often is salt, salty seasoning or a salty sauce (Such as Salt, Seasoning (e.g., Maggi, Knorr), Sauce (e.g., Soy sauce, Suya sauce, Fish sauce, Oyster sauce), and others) added to your household's cooking or preparing foods? | |  |  | |  | | |  | |  | |  |
| Do you add salt to any food while eating? | |  |  | |  | | |  | |  | |  |
| How often do you eat meals prepared outside of your home? (e.g., Restaurants, fast-food establishments, local roadside food vendors (Mama put)) | |  |  | |  | | |  | |  | |  |
| How often do you check food labels for Salt content when purchasing packaged foods? | |  |  | |  | | |  | |  | |  |
| How much salt or salty sauce do you think you consume? (Such as Salt, Seasoning (e.g., Maggi, Knorr), Sauce (e.g., Soy sauce, Suya sauce, Fish sauce, Oyster sauce), and others) | | Very low Low Moderate High    Very high Don’t know | | | | | | | | | |  |

| What milk do you usually use or drink, such as in hot & cold drinks or on cereal? (Including tea, coffee, hot milk, milkshakes, or cereal) | | | |
| --- | --- | --- | --- |
| Whole / full-fat milk |  | Semi-skimmed milk |  |
| Skimmed milk |  | Rarely/never use milk |  |
| Other (please write its name) | |  | |

**Other foods: Are there other foods and beverages you have eaten more than once a week in the past month?**

Yes No (If yes, please list below)

| Please tick how often you eat at least ONE portion of the following foods & drinks (Please only put one tick, but answer EVERY line) | | | | | | | |
| --- | --- | --- | --- | --- | --- | --- | --- |
| S/N | Food group/item | Usual Serving | Frequency | | | | |
|  |  |  | More than once/ day | Daily | 3-5 times a week | 1-2 times a week | Never/  Rarely |
| 1 |  |  |  |  |  |  |  |
| 2 |  |  |  |  |  |  |  |
| 3 |  |  |  |  |  |  |  |

**Time finished:  :**

**Table S2**: Categorizations of food groups in the FFQ corresponding to the FFQ question numbers.

| **S/N** | **Food group** | **List of Food items** |
| --- | --- | --- |
| 1 | Fruit | Fruit (q1) |
| 2 | Vegetable | Vegetables and Corn (maize) (q2) |
| 3 | Grains | Rice and Pasta (q3), Wheat products (q4), Fibre-rich breakfast cereals (q5), |
| 4 | Beans | Beans (q6) |
| 5 | Meat | Whole Meat (q7), White Meat (q7) |
| 6 | Processed meat | Processed Meats (q8) |
| 7 | Fish and seafoods | Fish (unbattered) or Whole fish (q10) |
| 8 | Eggs | Eggs (q11) |
| 9 | Fried or fast food | Fried or fast (takeaway) foods (q12) |
| 10 | Yam & potatoes | Potatoes and Yam (q13) |
| 11 | Soups | Fatty soups (14), Vegetable soups (q15), Draw soups (q16), Native soup (q17), |
| 12 | Stew | Stew (sauce) (q18) |
| 13 | Nuts & seeds | Nuts/seeds (q19) |
| 14 | Dessert and sweets | Ice cream, yoghurt, cheese, sweet, biscuits, cakes, chocolate, and others (q200 |
| 15 | Soft drinks | Fizzy (Soft) drinks and fruit juices (q22), Diet Non-alcoholic fizzy drinks (q23) |
| 16 | Alcoholic drinks | Alcoholic drinks (q24) |
| 17 | Tea and coffee | Tea and Coffee (q25) |
| 18 | Milk and milk drinks | Milk or milk-based beverages (q26) |
| 19 | Fats and oils | Fat and Oils (q21) |
| 20 | Salt and seasonings | Dietary salt (q27) |

FFQ: Food Frequency Questionnaire

**Table S3.** Bland-Altman analysis for the assessment of agreement between the food intake from the FFQ and 24DR.

| **Food groups (intakes/day)** | **Bias** | **Upper LOA** | **Lower LOA** | **% within LOA** |
| --- | --- | --- | --- | --- |
| Fruit | 0.03 | 1.21 | -1.16 | 97 |
| Vegetables | 0.02 | 0.66 | -0.62 | 100 |
| Grains | -0.03 | 0.46 | -0.52 | 100 |
| Beans and lentils | -0.02 | 0.58 | -0.62 | 100 |
| Meat | -0.06 | 0.65 | -0.77 | 100 |
| Processed meat | 0.02 | 0.38 | -0.34 | 98 |
| Fish | 0.08 | 1.40 | -1.23 | 97 |
| Eggs | 0.01 | 0.75 | -0.73 | 97 |
| Fried or fast food | -0.06 | 0.76 | -0.88 | 98 |
| Yam and potatoes | 0.06 | 1.21 | -1.20 | 98 |
| Soups | 0.02 | 0.48 | -0.43 | 98 |
| Stew | 0.03 | 0.84 | -0.77 | 100 |
| Nuts and seeds | 0.02 | 1.11 | -1.06 | 98 |
| Desserts and sweets | -0.01 | 1.01 | -1.03 | 97 |
| Soft drinks | 0.03 | 0.87 | -0.81 | 98 |
| Alcoholic drinks | -0.00 | 0.19 | -0.20 | 97 |
| Tea and coffee | 0.05 | 1.16 | -1.06 | 97 |
| Milk and milk drinks | -0.02 | 0.76 | -0.82 | 98 |
| Fats and oils | -1.73 | 0.73 | -4.18 | 84 |
| Salt and seasonings | -0.08 | 3.93 | -4.08 | 98 |

Bias: Mean difference between FFQ and 24DR, LOA: limits of agreement, %: percent


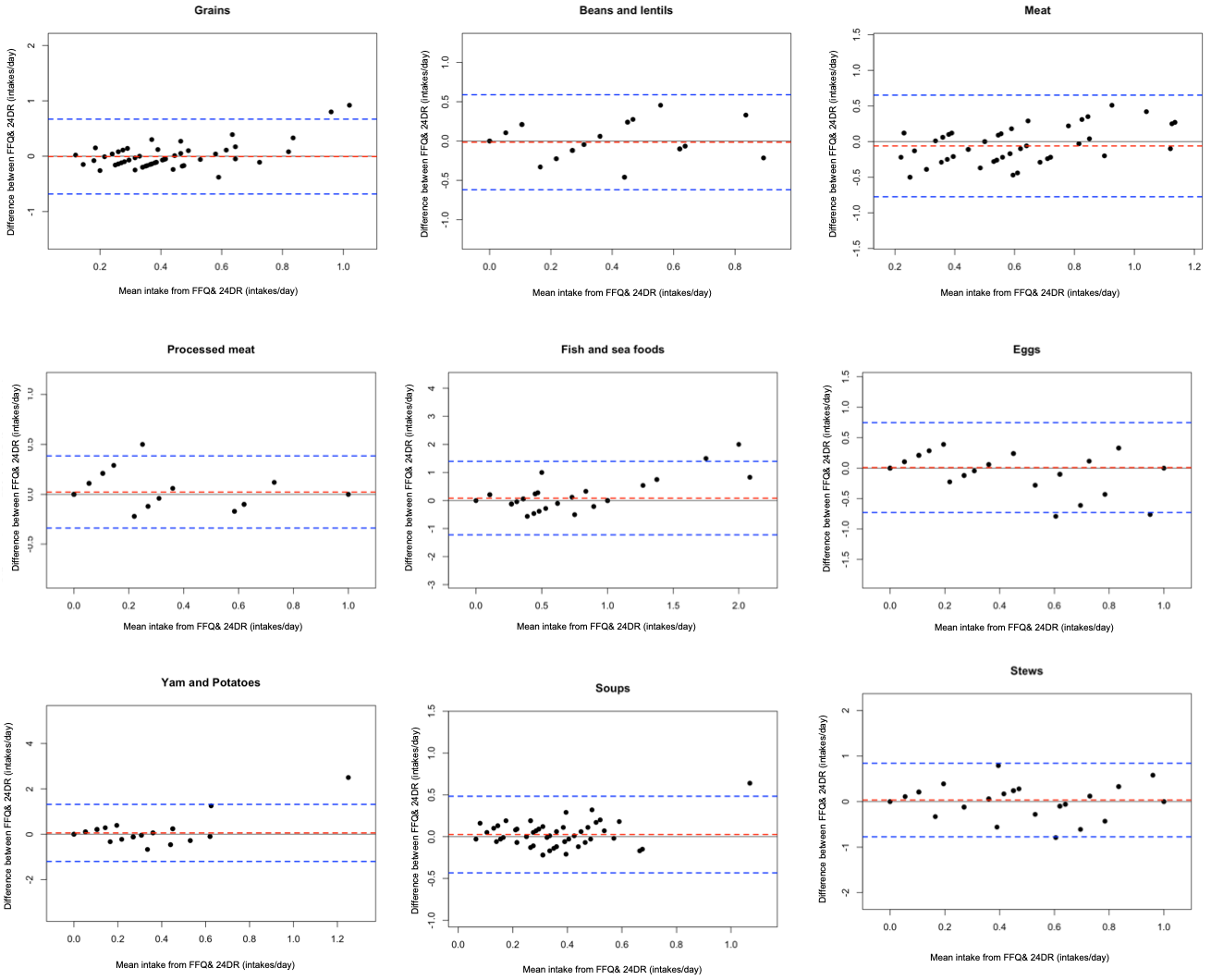


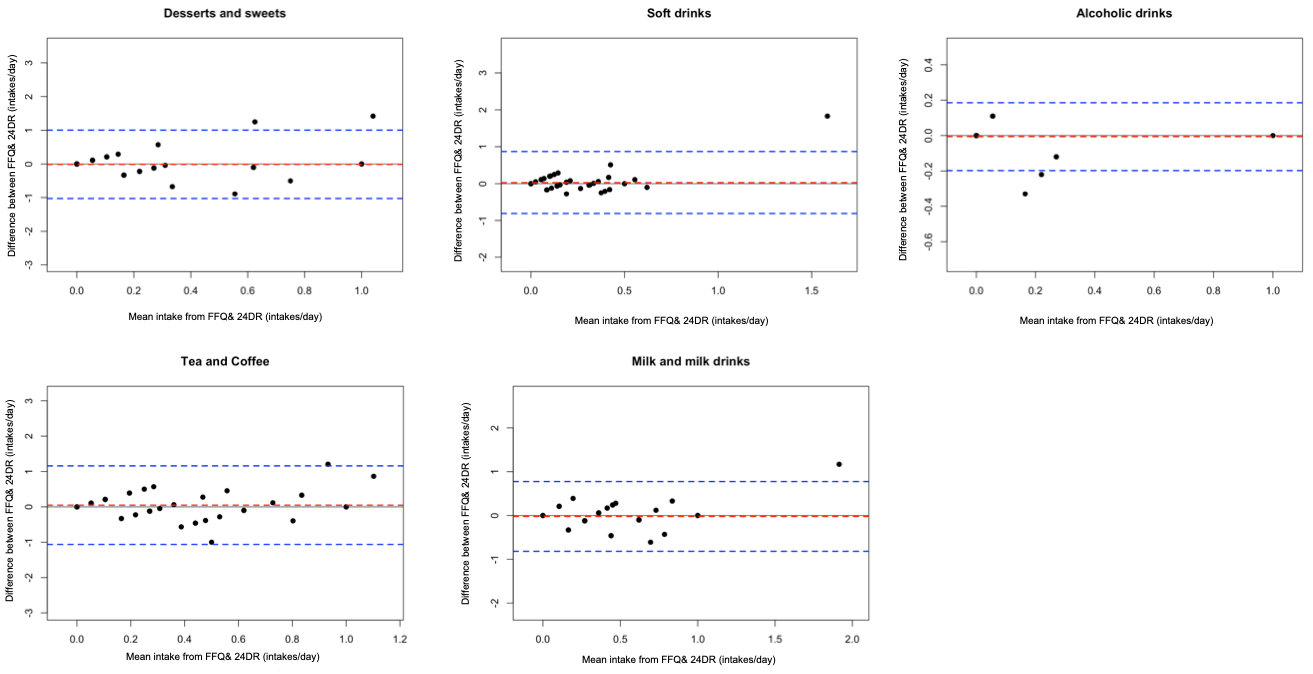


**Figure S1**. Bland-Altman plots related to food group intakes. Dashed red lines represent the mean difference, and dashed blue lines show the lower and upper 95% limits of agreement (*n*=58). 24DR: 24-hour dietary recall; FFQ: Food frequency questionnaire.
